# Measurement and Analysis of the Tracheobronchial Tree in Chinese Population Using Computed Tomography

**DOI:** 10.1371/journal.pone.0123177

**Published:** 2015-04-20

**Authors:** Weidong Mi, Changsheng Zhang, Hong Wang, Jiangbei Cao, Changtian Li, Li Yang, Fang Guo, Xianwang Wang, Tie Yang

**Affiliations:** 1 Anesthesia and Operation Center, Chinese PLA General Hospital, Beijing, 100853, China; 2 Department of Ultrasound, The Southern Building, Chinese PLA General Hospital, Beijing, 100853, China; 3 Department of Radiology, Chinese PLA General Hospital, Beijing, 100853, China; 4 Department of Anesthesia, The 309th hospital of Chinese PLA, Beijing, 100096, China; University of Pennsylvania, UNITED STATES

## Abstract

**Objective:**

To ascertain accurate measurements of, and the relationships between, the normative parameters of the tracheobronchial trees in the Chinese population using multi-slice spiral computed tomography (CT) and multi-planar reconstruction (MPR).

**Materials and Methods:**

Measurements were performed on 2107 patients who underwent thoracic CT scans in the PLA General Hospital. The lengths of the trachea and the main stem bronchi, and the sizes of the subcarinal angle were obtained through CT or MPR imaging. Multi-variance analyses were performed to detect potential correlations between obtained parameters.

**Results:**

The mean length of the trachea was 104.9 ± 13.4 mm (107.8 ± 13.2 mm for men and 101.4 ± 12.8 mm for women). The mean lengths of the right and left main stem bronchi were 13.6 ± 4.3 and 48.3 ± 6.5 mm, respectively. The right bronchus angle and the left bronchus angle were 34.9 and 42.5 degrees, respectively. Significant gender differences were found in all the parameters measured except for the angle of the right upper lobe bronchus. There are no statistically significant correlations among these parameters.

**Conclusions:**

The normal reference values and the likely ranges of distribution of the tracheobronchial trees in the Chinese population have been established. Significant gender differences exist in the dimensions of the trachea, with the exception of the Right upper bronchial angle (RUA).

## Introduction

The normal reference value and range of anthropometric variables differ among various races, and sometimes even among different ethnic groups within the same race[1]. For example, Mongolian patients tend to display smaller physical statures compared to those of Caucasian patients or Negroid patients. Therefore, the dimensions of the tracheobronchial tree may also differ from race to race. Since 1950s, there have been a number of studies on the dimensions of the tracheobronchial tree in the Western countries [2–6], and the data obtained from these studies formed the basis of the tracheobronchial tree dimensions in many textbooks.

Accurate anatomic knowledge of the trachea and the main bronchi is important not only in the field of pure anthropometry, but also in many other areas. Aside from direct applications in pulmonary physiology and thoracic surgery, such information has influences practices in anesthesiology. Ascertaining parameters of the tracheobronchial tree, such as the lengths, diameters, and angulations, helps optimizing surgical procedures such as intubation, reconstruction of the airway tree, and improving medical equipment, such as double-lumen endobronchial tube. Another clinical implication of this study is to guide the airway management for tracheal or bronchus tumor resection during the jet ventilation of interventional fiberoptic surgeries. It is usually critical at the time to make sure there is adequate ratio of jet catheter to the size of trachea or bronchus so that adequate oxygenation and ventilation is maintained but the risk of barotrauma is minimized [7, 8].

Furthermore, knowledge of a correlation, or lack thereof, among these parameters may reduce the need for unnecessary, expensive, or invasive diagnostic procedures by providing doctors with prior estimations of relevant parameters. For example, obtaining an accurate measurement of the tracheobronchial tree in a patient can ameliorate potential problems in airway management and improve patient care.

Despite of these benefits, precise measurements of the tracheobronchial tree in Chinese populations remain lacking. Previous studies on the dimensions of the tracheobronchial tree in Chinese populations were mostly performed by autopsy [2, 9–11], which deviates from normal physiological conditions, or chest radiography [3, 6, 12, 13], which lacks the accuracy of CT. As a result, these studies lack sufficient power to precise determinations of the dimensions of the airway tree in Chinese population. A few studies have utilized CT to measure the trachea; however, they are either focused on the growth of the trachea [14, 15] or certain parameters involved in intubation by double-lumen endotracheal tubes [16–19]. As a result these studies failed to provide adequate and accurate information about prominent features the airway tree, such as the length of the trachea and bronchi, the diameters of tracheal and bronchial lumens, and the angulation of the main stem bronchi. Furthermore, there were only several analyses on the relationship of parameters obtained [13, 20].

Here, we present a large scale study defining the anatomical features of the tracheobronchial tree in the population using multi-slice spiral CT and MPR. In addition, we analyzed potential correlations of parameters commonly used in defining the tracheobronchial tree. Our study provides a valid estimation measurement of the tracheobronchial tree in the Chinese population and should provide useful guidance for relevant clinical practice and medical device, especially the double-lumen tube, manufactures.

## Materials and Methods

### Cohort used in the study

From April 30^th^, 2011 to May 1^st^, 2013, 2700 patients were enrolled in this study from the PLA General Hospital, which provides medical service open to the entire Chinese population without restriction based on military service. The 2700 patients underwent thoracic CT scans in the Hospital; afterward their ages, heights, and weights were recorded under supervision. 200 patients were excluded from further study for meeting at least one of the following exclusion criteria: (1) non-Chinese; (2) younger than 18 or older than 90; (3) prior diagnosis of compulsive position, musculoskeletal deformity, or thoracic injury; (4) presence of hearing impairment or dysnoesia severe enough to preclude cooperation; and (5) a history of tracheobronchial surgery or trachea intubation. The remaining 2500 patients continued the study.

Prior to participation, all patients were fully informed the study and provided their informed, written consent. The study protocol was approved by the Ethics Committee of Chinese PLA General Hospital.

### Image Acquisition

All patients were trained to hold deep breath for at least 10s before the thorax CT scans during suspended end inspiration at total lung capacity. The arms were fully extended above the head. CT scans were performed using a SOMATOM Emotion 16 scanner (Siemens Healthcare, Forchheim, Germany) with the following setting: collimation, 1.2 × 16 mm; pitch, 0.95 mm; digital matrix, 512 × 512 pixels; tube potential, 130 kV. The tube current was chosen according to a dose modulation system (CARE Dose 4D, Siemens Medical Solutions). The acquisition time was selected based on the distance from the vocal cord to the diaphragm. The scanned CT images were uploaded to the local area network server of the Hospital and stored in DICOM format.

### Measurements

All CT images were measured using the image measurement software UniWeb Viewer 6.1.1152 (EBM Technologies, Inc.; Beijing, China). A lung window image sequence with a slice thickness of 1.5 mm was used for the measurements.

**Fig 1:**
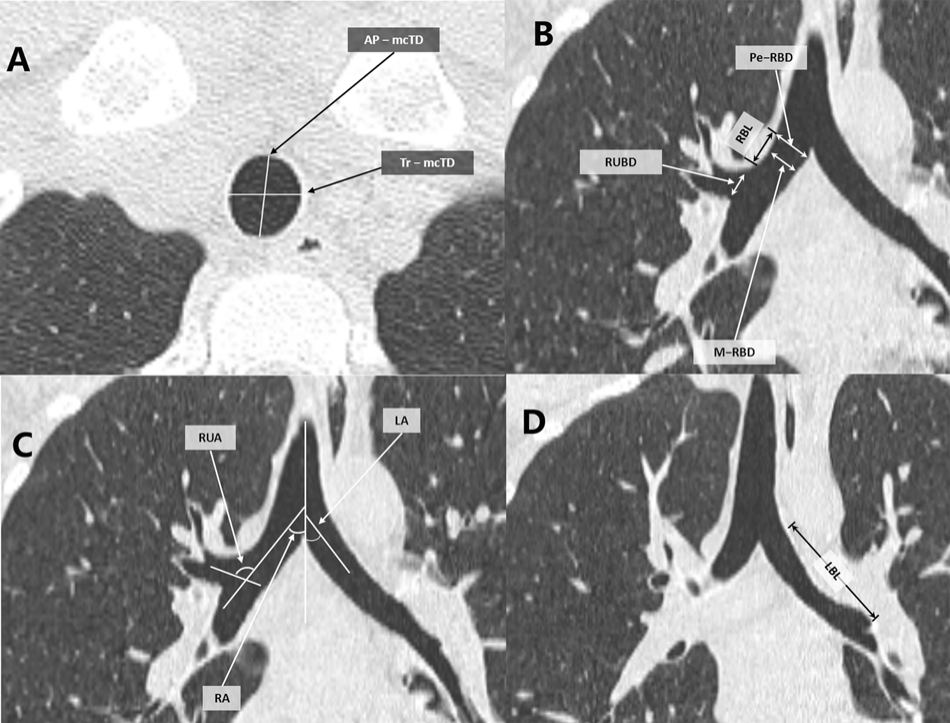
Measurements of the length, diameters and angles using CT scan and MPR. (A) AP-mcTD (Anteroposterior-Midclavicular tracheal diameter), Tr-mcTD (Transverse-Midclavicular tracheal diameter), (B) Pe-RBD (Proximal end-Right main bronchus internal diameter), RBL (Right main bronchus length), RUBD (Right upper bronchus internal diameter), M-RBD (Intermedius-Right main bronchus internal diameter), (C) LA (Left bronchial angle), RUA (Right upper bronchial angle), RA (Right bronchial angle), (D) LBL (Left main bronchus length).

The internal diameter of the trachea was first measured from the axial images. The internal diameter of the trachea was measured at two locations, one being just above the supraclavicular fossa (mcTD) to yield the anteroposterior (AP-) dimensions, and the other being in the middle of the whole trachea (mTD) to generate the transverse (Tr-) dimensions. These two locations were chosen because the supraclavicular fossa is easily recognizable and the middle point facilitates calculation. Subsequently, MPR was performed along the ordinate axes of the left and right bronchus at the carina plane of the axial images obtained through each CT scan. Tracheal length (TL) was measured as the distance between the lower border of the cricoid cartilage and the carina. The length of the right main stem bronchus (RBL) and the length of the left main stem bronchus (LBL) were measured as the distances between the tracheal bifurcation point and the point where RBL or LBL divides into the secondary bronchi, respectively. The length of the right middle lobe bronchus (RMBL) was measured as the distance between the far end of the orifice of the right upper lobe and the point where the RMBL divides into the right inferior lobe bronchus. The proximal end (Pe-) internal diameter and the intermedius diameter (M-) of the right (RBD) and left (LBD) main stem bronchus and the right middle lobe bronchus (RMBD) were measured perpendicular to its long axis on the MPR images, the same way as the diameter of the right upper lobe bronchial orifice. The angulation of the left bronchus (LA) was measured as the angle between the elongation of the distal end of the trachea and the proximal end of the left bronchus. The angulation of the right bronchus (RA) was measured as the angle between the elongation of the distal end of the trachea and the proximal end of the right bronchus. The angulation of the right upper lobe bronchus (RUA) was measured as the angle between the elongation of the right main stem bronchus and the right upper lobe. A level of 600 Hounsfield units and a width of 1600 Hounsfield units were used for the window settings. The results were shown in Fig 1.

### Statistical Analysis

SPSS version 13.0 (SPSS Inc.; Chicago, IL) was used for the statistical analysis. Each data point was measured three times in the presence of an anesthesiologist and a radiologist, and represented as the mean of three measurements. All data were presented using descriptive statistics (mean and standard deviation, and percentage). The unpaired Student’s *t* test was used to compare the gender-related differences in the tracheobronchial tree. The correlations between the tracheobronchial tree parameters and demographic parameters were analyzed using multiple regression analysis. The influence of gender was also examined by covariate analysis. The coefficient of variation (CV), which represents the extent of variability in relation to the mean of the population, is defined as the ratio of the standard deviation σ to the mean μ (Cv = σ/μ).

A *P* value <0.05 is considered statistically significant.

## Results

### Study Cohort

Of the 2500 subjects (1336 males, 1164 females) enrolled, 393 were excluded for meeting at least one of the following criteria: (1) presence of a tumor or other local process in the vicinity likely to affect the trachea and bronchus (202 cases); (2) presence of tracheo-bronchomegaly, tracheomalacia or bronchiectasis (39 cases); (3) presence of pneumothorax, hemathorax or massive hydrothorax (37 cases); (4) extensive consolidation or atelectasis (23 cases); (5) chronic tuberculosis (81 cases); and (6) unavailability of clearly identified borderline of the airway lumen on CT image (11 cases). Descriptive characteristics of the study cohort, including age, height, and weight, are summarized in Table 1. The study cohort consisted of 2107 subjects, with 1143 males and 964 females. The subjects ranged in age from 18 to 89 years, height from 145 to 192 cm, and weight from 40 to 115 kg. As expected, there are statistically significant differences in height and weight between the male and female subjects.

**Table 1:** Study Population Demographics*.

Variables	Male	Female	*P* Value
Patients	1143(54%)	964(46%)	
Age, yr	50.3±13.7	50.4±12.9	NS
Height, cm	171.5±5.8	160.4±5.2	<0.001
Weight, kg	70.2±10.8	59.8±9.3	<0.001

* Values are given as No. (%) or mean ±SD, unless otherwise indicated. NS = not significant.

### Tracheobronchial Measurements

The mean values of all the measured tracheobronchial parameters, including the length and diameter, were significantly higher for the male subjects than those of the female subjects, except for the LA, RA, and RUA (Table 2). The LA and RA values are significantly smaller in the male subjects compared to those of the female subjects, and no significant gender difference was found in the RUA (Table 2). The normal reference range of the tracheobronchial tree, as defined by mean value ± 95% standard deviation, was provided in Table 3. A statistically significant influence of gender on the relationships of the tracheal dimensions to height was detected from the univariate analysis that incorporates these variables as covariates. The AP-mcTD was larger than the Tr-mcTD in both sexes (*P* < 0.001). The diameter of the left main bronchus (Pe-LBD) was 1.5 mm larger on average than that of the bronchus intermedius (M-LBD) in the male subjects, and 1.3 mm in the female subjects; however, the diameter of the right main bronchus (Pe-RBD) was approximately the same as the bronchus intermedius (M-RBD). The mean subcarinal angle, defined as the angle of the right bronchus angulation plus the angulation of the left bronchus, was significantly larger in the female subjects than in the male subjects (80.1 ± 13.4 degrees *vs*. 75.1 ± 13.4 degrees). The RA was larger than the LA in 20.6% of the study cohort (Table 4). In addition, the frequency of pre-carinal take-off of the RUL bronchus was 8.4‰ in the Chinese population.

**Table 2:** Tracheobronchial Parameters.

Variables	Male	Female	Combined	*P* Value
**Diameters(mm)**				
AP-mcTD	19.9±2.4	15.0±2.0	17.7±3.3	<0.001
Tr-mcTD	16.8±2.1	14.6±1.7	15.8±2.2	<0.001
AP-mTD	19.0±2.3	14.9±2.1	17.1±3.0	<0.001
Tr-mTD	17.1±2.6	14.9±2.0	16.1±2.5	<0.001
Pe-LBD	13.1±1.7	11.3±1.6	12.3±1.9	<0.001
M-LBD	11.6±1.6	10.0±1.5	10.9±1.7	<0.001
Pe-RBD	14.1±1.9	12.3±1.7	13.3±2.0	<0.001
M-RBD	14.1±2.0	12.2±1.8	13.2±2.1	<0.001
RUBD	9.9±1.8	8.8±1.7	9.4±1.8	<0.001
Pe-RMBD	12.9±1.8	11.3±1.6	12.2±1.9	<0.001
M-RMBD	11.0±1.8	9.5±1.5	10.3±1.8	<0.001
**Length (mm)**				
TL	107.8±13.2	101.4±12.8	104.9±13.4	<0.001
LBL	50.0±6.3	46.2±6.0	48.3±6.5	<0.001
RBL	14.1±4.5	12.9±4.0	13.6±4.3	<0.001
RMBL	32.3±6.7	29.0±6.4	30.8±6.8	<0.001
**Angles (in degrees from tracheal axis)**				
LA	41.2±8.9	44.1±8.6	42.5±8.9	<0.001
RA	34.0±7.8	36.0±8.1	34.9±8.0	<0.001
RUA	73.7±15.0	73.8±15.2	73.7±15.1	NS

Values are given as mean ±SD. NS = not significant. AP-mcTD: Anteroposterior Midclavicular tracheal diameter; Tr-mcTD: Transverse Midclavicular tracheal diameter; AP-mTD: Anteroposterior Middle tracheal diameter; Tr-mTD: Transverse Middle tracheal diameter; Pe-LBD: Proximal end Left main bronchus internal diameter; M-LBD: Intermedius Left main bronchus internal diameter; Pe-RBD: Proximal end Right main bronchus internal diameter; M-RBD: Intermedius Right main bronchus internal diameter; RUBD: Right upper bronchus internal diameter; Pe-RMBD: Proximal end Right middle bronchus internal diameter; M-RMBD: Intermedius Right middle bronchus internal diameter; TL: Tracheal length; LBL: Left main bronchus length; RBL: Right main bronchus length; RMBL: Right middle bronchus length; LA: Left bronchial angle; RA: Right bronchial angle; RUA: Right upper bronchial angle.

**Table 3:** Normal Reference Range of Tracheobronchial Tree in Chinese People.

Variables	Male		Female	
	lower bound	upper bound	lower bound	upper bound
AP-mcTD (mm)	15.1	24.7	11.1	18.9
Tr-mcTD (mm)	12.6	21.0	11.3	17.9
AP-mTD (mm)	14.4	23.5	10.8	19.0
Tr-mTD (mm)	12.0	22.1	11.1	18.8
Pe-LBD (mm)	9.7	16.5	8.1	14.5
M-LBD (mm)	8.5	14.6	7.0	13.0
Pe-RBD (mm)	10.3	17.9	9.0	15.6
M-RBD (mm)	10.2	18.0	8.7	15.7
RUBD (mm)	6.4	13.3	5.5	12.2
Pe-RMBD (mm)	9.4	16.4	8.2	14.4
mRMBD (mm)	7.6	14.5	6.6	12.4
TL (mm)	81.9	133.7	76.3	126.5
LBL (mm)	37.5	62.4	34.5	58.0
RBL (mm)	5.3	22.9	5.1	20.7
RMBL (mm)	19.1	45.4	16.4	41.6
LA (°)	23.7	58.6	27.2	61.1
RA(°)	18.7	49.2	20.1	51.8
RUA(°)	44.3	103.1	44.0	103.5

**Table 4:** Comparison Between Right Bronchial Angle and Left Bronchial Angle.

Comparison	Number of people		Combined
	Male	Female	
RA > LA	262	171	433(20.6%)
RA = LA	10	10	20(0.95%)
RA < LA	871	783	1654(78.5%)

The RBL had the largest coefficient of variation (31.70% in the male subjects and 30.96% in the female subjects) among all the parameters, suggesting a large individual variation of the RBL length. The coefficients of variations for RMBL, LA, RA, and RUA were smaller than that of the RBL. The coefficients of variations of other parameters were relatively small, indicating a smaller individual variation of the rest parameters in the Chinese population.

### Interrelationship Analysis

**Fig 2:**
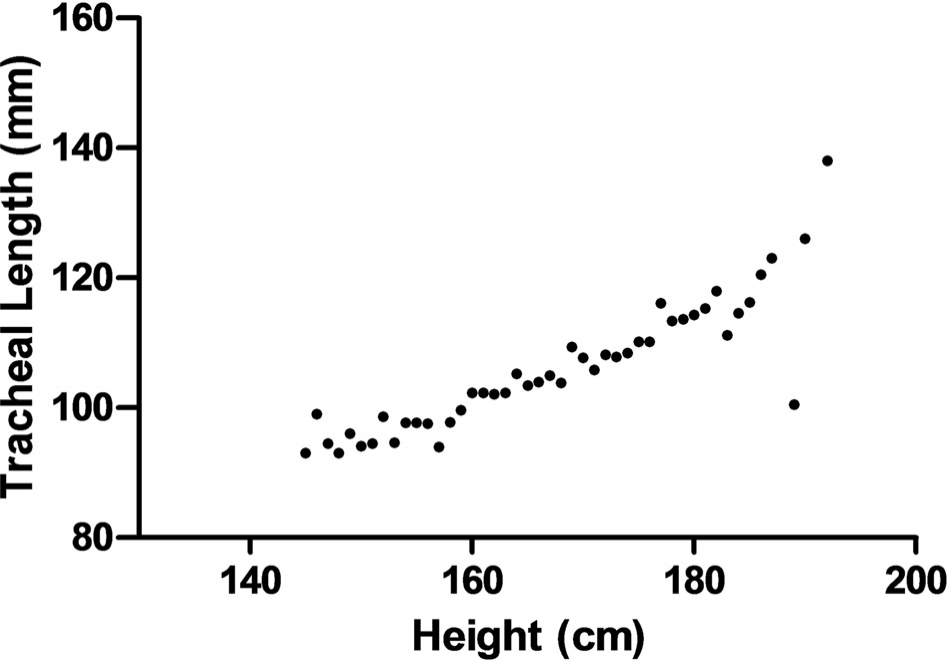
The scatterplot of the average TL (Tracheal length) and Height. (A) Male, (B) Female.

Extrapulmonary relationships: the dimensions of the airway were poorly correlated with the height, weight, and age. The correlation coefficient between the trachea length and the height was 0.378 for all subjects. The AP-mcTD had a statistically significant correlation with the height and age (*r* = 0.629), but not weight. In addition, a suggestive positive trend was found between the average tracheal length and the height (Fig 2, the linear regression equation for both gender is *TL (mm) = * 0.665*Height (cm) -5.226, *TL (mm) = * 0.729*Height (cm) -16.837[male], *TL (mm) = * 0.697*Height (cm) -9.539[female]).

Intrapulmonary relationships: The correlation coefficient between the Pe-LBD and the AP-mTD was 0.498. The correlation coefficient between the AP-mcTD and the Tr-mcTD was 0.579. The AP-mTD and Tr-mTD together were a statistically significant predictor of the Pe-RBD (*r* = 0.560). Lastly, there was significant correlation between the Pe-RBD and Pe-LBD (*r* = 0.671).

Regression analysis was performed to ascertain correlations that can be expressed in mathematical equations to predict the diameters for individual subjects; however, the predictive power and the coefficient of determinations of such equations were not good enough to demonstrate the possibility of the underlying correlation (Table 5). The correlation coefficients of the above extrapulmonary and intrapulmonary relationships were much lower when the outputs are organized by gender groups, although these relationships were statistically significant.

**Table 5:** Correlation Between Parameters and the Line of Best Fit.

Dependent Variable	Predictive Factors	*r*/*r* ^2^ value	P value	Line of best fit
TL	Height	0.378/0.143	<0.001	-
	Weight	0.108/0.012	<0.001	-
AP-mcTD	Height & Age	0.629/0.396	<0.001	AP-mcTD(mm) = 0.265×Height(cm)+0.048×Age(yr)-28.933
AP-mcTD	Tr-mcTD	0.579/0.335	<0.000	AP-mcTD(mm) = 0.860×Tr-mcTD(mm)+4.094
Pe-LBD	AP-mTD	0.490/0.240	<0.001	-
Pe-LBD	AP-mTD & Tr-mTD	0.573/0.329	<0.001	Pe-LBD(mm) = 0.248×AP-mTD+0.228×Tr-mTD+4.377
Pe-RBD	AP-mTD & Tr-mTD	0.560/0.314	<0.001	Pe-RBD(mm) = 0.213×AP-mTD+0.292×Tr-mTD+4.923
Pe-RBD	Pe-LBD	0.671/0.450	<0.001	Pe-RBD(mm) = 0.719×Pe-LBD+4.450

### Age-Related Dimensions Analysis

**Fig 3:**
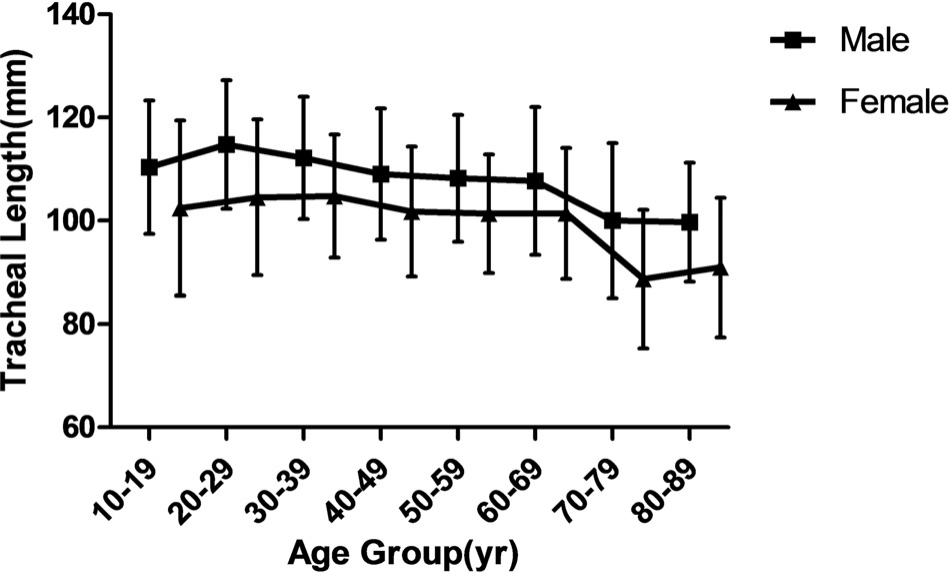
Lengths of trachea from subjects with age 18 to 90. Values for each 10 year period were averaged.

**Fig 4:**
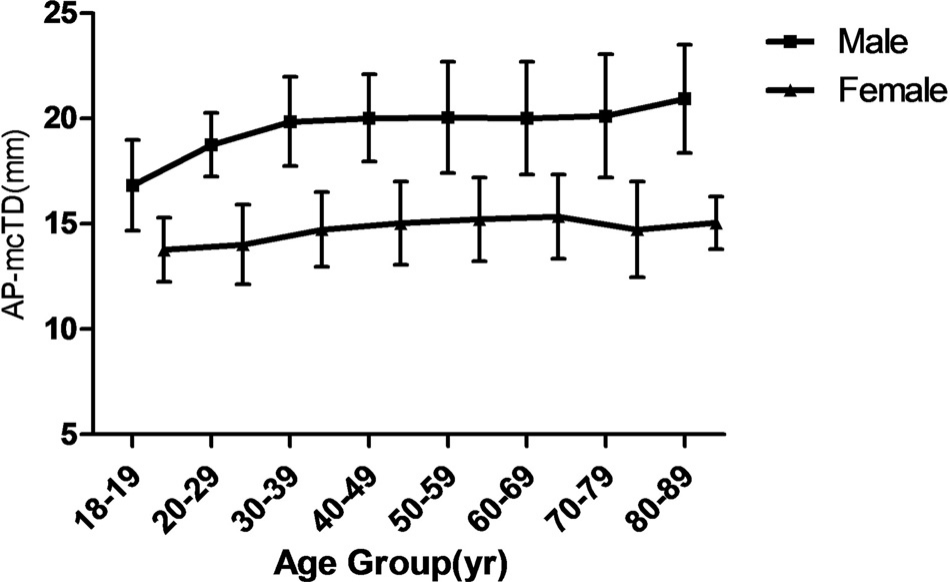
Mean AP-mcTD (Anteroposterior-Midclavicular tracheal diameter) of trachea from subjects with age 18 to 90. Values for each 10 year period were averaged.

**Fig 5:**
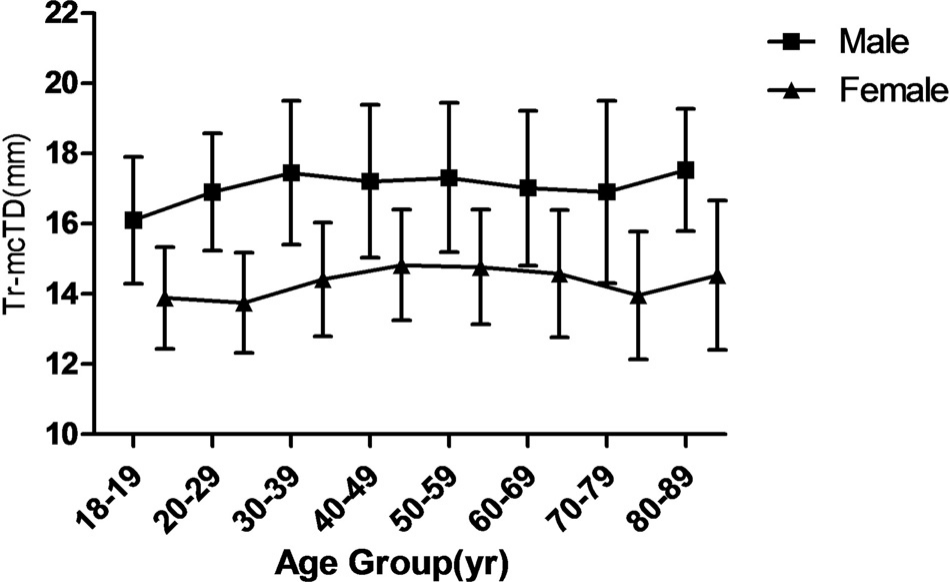
Mean Tr-mcTD (Transverse-Midclavicular tracheal diameter) of trachea from subjects with age 18 to 90. Values for each 10 year period were averaged.

The tracheal lengths (TL) were significantly smaller in the two oldest age groups than in the other six age groups (*P* < 0.001), as shown in Fig 3 which plots the TL against age in both the male and female subjects. The AP-mcTD distribution curve against age in both the male and female subjects is presented in Fig 4. The Tr-mcTD distribution curve against age in both the male and female subjects is presented in Fig 5. The mean values of the AP-mcTD and the Tr-mcTD for each age group and either gender are presented in Table 6.

**Table 6:** Mean Anteroposterior and Transverse Midclavicular Tracheal Diameters in Normal Subjects by Gender and Age.

Age groups yr.	Male			Female		
	No. of Subjects	mcTD diameter(mm): Mean±SD		No. of Subjects	mcTD diameter(mm): Mean±SD	
		Anteroposterior	Transverse		Anteroposterior	Transverse
10–19	12	16.8±2.2	15.7±1.8	17	13.8±1.5	13.9±1.5
20–29	88	18.8±1.5	16.5±1.7	50	14.0±1.9	13.8±1.4
30–39	135	19.9±2.1	17.1±2.1	109	14.7±1.8	14.4±1.6
40–49	282	20.0±2.1	16.8±2.2	256	15.0±2.0	14.8±1.6
50–59	329	20.1±2.6	16.9±2.1	302	15.2±2.0	14.8±1.6
60–69	226	20.0±2.7	16.6±2.2	180	15.4±2.0	14.6±1.8
70–79	51	20.1±2.9	16.5±2.6	42	14.7±2.3	14.0±1.8
80–89	20	20.9±2.6	17.1±1.7	8	15.1±1.2	14.5±2.1
Total	1143	20.0±2.4	16.8±2.1	964	15.0±2.0	14.6±1.7

## Discussion

In the present study, we measured the lengths of the trachea in a large cohort of Chinese and found that the mean tracheal length was 104.9 ± 13.4 mm. The male subjects on average have a longer trachea than the female subjects (107.8 ± 13.2 mm *vs*. 101.4 ± 12.8 mm). The length of the left main stem bronchus was 2.5 fold longer than that of the right main stem bronchus [48.3 ± 6.5 mm (range, 22.9–60.9 mm) *vs*. 13.6 ± 4.3 mm (range, 2.6–25.9 mm)]. The mean AP-mcTD was 17.7 ± 3.3 mm, while the mean Tr-mcTD was 15.8 ± 2.2 mm. The right main stem bronchus branched off the trachea at an average angle of 34.0 degrees in the male subjects and at an average angle of 36.0 degrees in the female subjects. The average angle between the left main stem bronchus and the trachea is 41.2 degree for the male subjects and 44.1 degree for the female subjects. The average angle between the left main stem bronchus and the trachea is 42.5 degree for the male and female subjects combined. The variations between the male and the female subjects are all statistically significant.

The results above indicate: (1) the male subjects have larger diameters and length of the tracheobronchial tree, while the the female subjects have larger main stem bronchial angles and subcarinal angles; (2) there is a strong positive correlation between the average tracheal length and body height, and there is also a positive trend between age and AP-mcTD; (3) the large individual variations in the parameters relating to the tracheal dimension precluded a simple math equation capable of predicting TL, AP-mcTD, Pe-RBD and Pe-LBD. (4) TL, RBL, LBL, TD, RA and LA have clinical applications and are further discussed below.

### Tracheal length

Our results on the length of the trachea correlate well with parameters obtained by other methods as published on Chinese textbooks [21, 22], but differ from sources [10, 11, 15, 23, 24] obtained from other countries. This results caution against borrowing data across ethnic groups without validation.

To investigate whether gender influences the tracheal dimensions, a univariate analysis incorporating sex as covariates was performed. We found that gender had a significant influence on the TL, and the average TL at any given body height was slightly longer in females (*P* = 0.03). Gender has a significant influence on the TL. The influence may be partially attributable to the gender difference in height. However, our data suggest that gender difference other than height may contribute to the average TL difference between the male and female subjects. First, the correlation between the TL and the height was poor (*r* = 0.38). Some previous studies [11, 14, 25] have reported significant correlations between the TL and the height only in young subjects during growth period. Second, there are large individual variations of the TL even among subjects of the same height. Further studies are needed to elucidate other potential factors underlying the gender differences of the TL.

Our study also revealed a decrease of the TL in subjects 70 years or older. This may results from a reduction of fibrous tissue in this age group. In addition, the trachea is much more vertical on lateral projection in youth than in old age [24]. In conclusion, height, weight, and age by themselves are not reliable factors in predicting the TL.

### Length of the Main Bronchi

Our result on the length of the right main stem bronchi is 32% smaller than a previous textbook [22] on Chinese. Our results on the length of the main stem also differ from those from the findings from other countries [11, 23, 26] again highlighting the importance of confirming anatomical measurements within individual ethnic groups.

The large coefficient of variance found among the lengths of the right bronchi (~31%), together with the finding that the incidence of takeoff of the right upper lobe from the trachea (8.4‰) was higher than previously reported [26], suggests a large individual variation in the length of the right main stem bronchus.

Our study also has implication on clinical practices. It has been noted that Broncho-Cath double lumen tubes (Mallinckrodt, Athlone, Ireland) pose a risk to patients when the tube length exceed the lengths of the right bronchi by 10 mm, which may cause airway trauma and rupture of the membranous part of the trachea [27]. Adopting the safety margin described by Benumof *et al*.[28], we found that 21.4% of the enrolled subjects (451) were in risk because their lengths of the right bronchi are 10 mm shorter than the tubes. This percentage is much higher than that reported by Benumof *et al*. (11%) [28]. If these patients were intubated a right-sided double-lumen endobronchial tube, the right upper orifice may easily be obstructed by the endobronchial balloon. In addition, for 20 subjects (~1%), the length of the left bronchus is shorter than 32 mm, the length of the left bronchus tube. Using the tube in these subjects would have resulted in airway obstruction during one-lung ventilation by deep insertion of the left lumen tip. Therefore, our study predicts that if a Mallinckrodt right-sided tube is used, right upper lobe obstruction would have occurred at a much higher rate in Chinese patients compared to the Manufacturer's estimation, even under a condition where the endobronchial cuff is right below the tracheal bifurcation. Thus, our findings strongly support three clinical practice suggestions: (1) the left sided double-lumen tube should be used whenever possible; (2) anesthesiologists should review and measure the thorax CT image before intubation in order to identify the airway tree morphology and choose a best-fit endobronchial tube; (3) Fiberoptic bronchoscope is recommended to confirm proper placement of endobronchial tube.

### Diameter of the Trachea and Main Stem Bronchi

Our finding that the AP-mcTD was about 1.9 mm larger than Tr-mcTD is consistent with previous publications [6, 15, 18], but correlations between AP-mcTD and both body height and age (*r* = 0.629) were not large enough compared to an satisfied *r* > 0.9, even if *P* < 0.01. The right main stem bronchus was 1.0 mm wider than the left main stem bronchus in our study, which is in good agreement with the study by Seymour [9]. However, tracheobronchial size and shape varies with body position, intraluminal pressure [29] and respiratory phase [30], and may undergo considerable change from moment to moment [15]. The individual variation and the difference in the degree of deep breath during CT scan presumably contribute to the weak correlations between tracheal diameter or body characteristics. As shown in Table 5, there was a statistically significant correlation between the mTD and the proximal end diameters of the bronchi, and the correlation coefficient between the diameters of the two bronchi obtained in this study was similar to the previously reported value by Hampton *et al*. (0.754) [31]. In addition, the results in Figs 4 and 5 could be partly attributable to the atrophy of the mucosa and the reduction of the flexibility of the trachea.

### Angles of the Main Bronchi and Subcarinal

The angles of the main bronchi and subcarinal we observed correspond closely to those reported previously [21]. We found that the average subcarinal angle measured in the female subjects was greater than that in the male subjects, in contrary to a previous cadaveric observation [11]. This discrepancy may be explained by the observation that lungs grow more transversally than downwards in females before the chest wall becomes rigid and the diaphragmatic muscle is stronger in males. In addition, a cadaver differs from a living body, due to the relative position of active and inactive diaphragm as observed by Fearson *et al*. [32].

The angle of the right main stem bronchus was larger than the angle of the left main stem bronchus in 20.6% of the subjects, whereas in Grey’s Anatomy [33] the angle of the right main stem is presumed to be consistently smaller. Our study indicates that in one fifth of the Chinese population, the left main stem bronchus is more vertical and the lodgment of a foreign body would be more common in the left bronchus than in the right bronchus.

In summary, we obtained the normative values ranges of the tracheobronchial tree for Chinese people through a large-scale study using CT. We further analyzed the relationships between the principle parameters defining the tracheobronchial tree. Our results provide a basis for improving clinical practice in the area of DLT intubation. Our results further revealed the unique aspects of the trachea anatomy in Chinese people. Our results indicate certain parameters such as TL, LBL, Tr-mcTD have a narrow distribution, whereas other parameters, such as RMBL, LA, RA, and RUA demonstrate large individual variability among Chinese people. Despite the strong correlation between the height and length of TL, no accurate and reliable equations were found for predicting the complete tracheobronchial dimensions through height, age, and gender alone or in combination. Computed tomography and bronchoscopy should remain the most reliable methods for accurately determining the airway dimensions.
