# Correction: Measurement and Analysis of the Tracheobronchial Tree in Chinese Population Using Computed Tomography

**DOI:** 10.1371/journal.pone.0130239

**Published:** 2015-06-03

**Authors:** Changsheng Zhang, Hong Wang, Jiangbei Cao, Changtian Li, Weidong Mi, Li Yang, Fang Guo, Xianwang Wang, Tie Yang

The authors are listed out of order. Please view the correct author order, affiliations, and citation here:

Changsheng Zhang^1☯^, Hong Wang^1☯^, Jiangbei Cao^1^, Changtian Li^2^, Weidong Mi^1^*, Li Yang^3^, Fang Guo^1^, Xianwang Wang^4^, Tie Yang^3^

1 Anesthesia and Operation Center, Chinese PLA General Hospital, Beijing, 100853, China, 2 Department of Ultrasound, The Southern Building, Chinese PLA General Hospital, Beijing, 100853, China, 3 Department of Radiology, Chinese PLA General Hospital, Beijing, 100853, China, 4 Department of Anesthesia, The 309th hospital of Chinese PLA, Beijing, 100096, China

☯ These authors contributed equally to this work.

* plamzk@126.com

Zhang C, Wang H, Cao J, Li C, Mi W, Yang L, et al. (2015) Measurement and Analysis of the Tracheobronchial Tree in Chinese Population Using Computed Tomography. PLoS ONE 10(4): e0123177. doi:10.1371/journal.pone.0123177
